# Characterization of Surfactant Spheroidal Micelle Structure for Pharmaceutical Applications: A Novel Analytical Framework

**DOI:** 10.3390/pharmaceutics16050604

**Published:** 2024-04-29

**Authors:** Liberato De Caro, Thibaud Stoll, Arnaud Grandeury, Fabia Gozzo, Cinzia Giannini

**Affiliations:** 1 Istituto di Cristallografia, Consiglio Nazionale delle Ricerche, Via Amendola 122/O, 70125 Bari, Italy; cinzia.giannini@cnr.it; 2 Excelsus Structural Solutions (Swiss) AG, Park Innovaare, Parkstrasse 1, 5234 Villigen, Switzerland; thibaud.stoll@excelsus2s.com (T.S.); fabia.gozzo@excelsus2s.com (F.G.); 3 Novartis Pharma AG, Technical Research and Development, Material Science, Novartis Campus, Virchow 6.3.231, 4056 Basel, Switzerland; arnaud.grandeury@novartis.com

**Keywords:** drug delivery systems, surfactant, small angle X-ray scattering, micelles

## Abstract

We introduce an innovative theoretical framework tailored for the analysis of Pair Distribution Function (PDF) data derived from Small-Angle X-ray Scattering (SAXS) measurements of core-shell micelles. The new approach involves the exploitation of the first derivative of the PDF and the derivation of analytical equations to solve the core-shell micelle structure under the hypothesis of a spheroidal shape. These analytical equations enable us to determine the micelle’s aggregation number, degree of ellipticity, and contrast in electron density between the core-shell and shell-buffer regions after having determined the whole micelle size and its shell size from the analysis of the first derivative of the PDF. We have formulated an overdetermined system of analytical equations based on the unknowns that characterize the micelle structure. This allows us to establish a Figure of Merit, which is utilized to identify the most reliable solution within the system of equations.

## 1. Introduction

Surfactants are amphiphilic molecules, consisting of a hydrophilic “head” and a hydrophobic “tail”. They are typically classified according to the electric charge(s)—if any—of their heads, as non-ionic, anionic, cationic, or amphoteric. The hydrophobic tail, typically comprising an alkyl chain of 8–22 carbons, can be either lipophilic or lipophobic, as seen in the presence of hydrocarbon or fluorocarbon groups. Recent studies have also highlighted the importance of the “linker”, connecting the head with the tail, in influencing surfactant properties [1,2]. Given this amphiphilic nature, many surfactants, when present in aqueous solutions above a “critical micelle concentration” (CMC), can self-assemble into supramolecular structures known as micelles.

The size and shape of a micelle are influenced by various factors, such as the structure of the surfactant (head, tail, and linker), its concentration, and the environmental conditions, like temperature, pH, and composition of the aqueous solution. Typically, micelles can adopt an ellipsoidal shape. Spherical micelles are observed in solutions with surfactants that have a relatively balanced structure of hydrophilic and hydrophobic regions. In micelles, the hydrophobic tails of the surfactant molecules are oriented toward the center of the sphere, creating a hydrophobic core, while the hydrophilic heads face the surrounding aqueous environment. Ellipsoidal or rod-like micelles form when surfactant molecules possess pronounced elongation or asymmetry in their molecular structure. Such molecules may align to create elongated micelles with an elliptical or rod-like shape. Additionally, certain surfactant systems may exhibit transitions between different micellar shapes under varying conditions [1].

Micelles find extensive use in pharmaceutical applications due to their ability to stabilize, solubilize, and improve the bioavailability of a wide variety of active ingredients, such as small molecules, biologics, vaccines, and mRNA. For this purpose, the characterization of the micelle size and shape and its dynamic behavior under various conditions is of crucial importance [2].

Furthermore, there is an industry-wide need to develop novel surfactants with enhanced stabilization properties, safety profiles, and improved chemical stability. For instance, for biologics, to date mostly polysorbates (PS20/80) and poloxamer 188 are used in commercial products. However, both surfactant families suffer from severe degradation and impurities of raw materials, which frequently increase the risk of particle generation, chemical protein degradation, and potential adverse immune reactions [3]. An enhanced method for micelle characterization could aid in understanding the relationship between the micelle properties and the structure of the constituting surfactant monomers. This could lead to predictive models and provide a basis for a rational design of surfactants (head, tail, and linker) based on the desired properties of micelles for a specific application.

In this framework, pharmaceutical companies have begun incorporating small-angle X-ray scattering (SAXS) technique analysis into their workflows, finding it powerful for characterizing various biopharmaceuticals. Indeed, SAXS holds a distinctive position among the array of methods available for biological research. Measurements are conducted in a solution, making SAXS a potent tool for analyzing macromolecules in their quasi-native environment. The sample can be easily examined by changing in-situ various variables such as temperature, pH, time, light, mechanical stress, etc. From the scattering data, overall molecular size/shape, and weight, along with the degree of compactness and flexibility, can be readily determined. In addition to this analytical information, the scattering profiles also encompass structural details. In many cases, it is feasible to directly reconstruct the overall shape of the macromolecule without any prior knowledge about the system, albeit at low resolution (~1–2 nm).

Two main factors have contributed to the increased popularity of solution SAXS: (i) improved X-ray sources (both synchrotrons and laboratory sources) combined with novel advanced detectors have significantly reduced the required sample volume. Robotic systems for sample handling have, furthermore, substantially improved the efficiency and user-friendliness of data acquisition. Nowadays, even challenging samples, difficult to obtain in large amounts, can be examined within minutes. (ii) Novel methods for data analysis [4] and related software tools have become available to facilitate the interpretation of scattering patterns from biological systems. Most of the available software allows us to calculate theoretical scattering SAXS curves from macromolecular models to be compared with the experimental data, generate the Pair Distribution Function (PDF) from the measured SAXS intensities, and determine size/shape models of the investigated scattering samples. Here is an incomplete list of SAXS software available for users:

- ATSAS package [5], which includes various programs (PRIMUS, GNOM,…) for SAXS data analysis and structure modeling, provides information such as distance distribution function, gyration radius, molecular weight, flexibility, etc.
- FoXS, FoXSDock, and MultiFoXS [6,7] for rapid calculation of low-resolution scattering functions based on structural models.
- SASView [7], an open-source software designed for scattering analysis with a focus on user-friendliness and data visualization.
- SCATTER [8], a program for the analysis, modeling, and fitting of 1D and 2D SAXS data of non-ordered, partially ordered, or fully ordered nano- and mesoscale structures.
- SAXSMoW [9,10], an online calculator of the molecular weight of proteins in dilute solution from experimental SAXS data.

It is important to note that the choice of software often depends on specific analysis needs and user preferences, as different programs may offer varied functionalities and approaches to SAXS analysis. User-friendly options have been developed over the past decade for fast, streamlined data analysis, but care must be taken in both processing the data and understanding the results because, when the outputs of different software are compared, sometimes the obtained structural solution might be software-dependent.

In this framework, we have recently investigated by SAXS the structure and functionality of a D-α-tocopherol polyethylene glycol 1000 succinate (VitE-TPGS) system of micelles, without and with an addition of a Poorly Soluble Compound (PSC), assuming a spherical-shaped hydrophobic inner core and an outer hydrated hydrophilic shell [4]. A simple two-dimensional/two-components theoretical model was developed to describe the SAXS data and related Pair Distribution Function (PDF), collected from the VitE-TPGS micelles with and without the PSC Eltrombopag. Under the spherical-shape assumption, the quantitative analysis showed that the PSC molecules localized mostly out of the micelle’s core, thus still in interaction with buffer liquid, localized near the middle part of the VitE-TPGS molecule, bounded at the succinate linker and benzodihydropyran rings position, which connect the hydrophobic aliphatic chain of the α-tocopherol part to the hydrophilic PEG chain. 

In the case of dodecyl phosphocholine micelles, using various core–shell models consisting of a hydrophobic core and a shell representing hydrated polar headgroups, it was shown that a spherical model incorporating a core–shell mixed region used to describe non-ideal interfaces could account for the changes in the size and shape of the scattering profiles [11]. These non-ideal core-shell and shell-solvent interfaces were also implemented in our work [4], where we convolved the theoretical PDF profiles with a gaussian function. Clearly, a further fitting parameter was introduced in the modeling [4,11], making the model equivalent, in terms of total number of parameters, to that obtainable assuming spheroidal micelles, i.e., non-perfect spherical micelles but prolate/oblate ellipsoids with the polar axis longer/shorter with respect to the equatorial axis. In other words, the obtained results on the core/shell sizes, their electron density contrasts, and the actual shape of the micelles, as already underlined, are clearly model-dependent.

For this reason, in the present work we have developed a new approach based on analytical equations to solve the unknown structure of two-component core-shell micelles directly from the PDF analysis, under a general assumption of a spheroidal shape for the micelles, hence admitting departures from pure spheric shapes. In particular, we propose a new method of analysis of the PDF first derivative to obtain an immediate and reliable graphical estimate of the core and shell sizes and make them as much as possible independent of any fitting software used for SAXS/PDF simulations.

In the following: (i) we discuss how to graphically estimate—from the PDF first derivative—the core and shell sizes of the micelle under study; (ii) we derive analytical equations useful both for determining the core-shell and shell-buffer electron density contrasts, starting from the core and shell sizes derived graphically; (iii) we apply the new proposed graphical-analytical approach, whose mathematical details are reported in Appendix A, to micelles formed by different surfactants that have been previously characterized, namely PS20 [12], DPC and SDS [13], and VitE-TPGS with and without a PSC [4]. This comparison with literature data helped us validate the new graphical-analytical method and demonstrate its potentiality compared to the conventional approach based on the current SAXS/PDF simulation software [5,6,7,8,9,10].

## 2. The New Graphical-Analytical Approach

**Figure 1:**
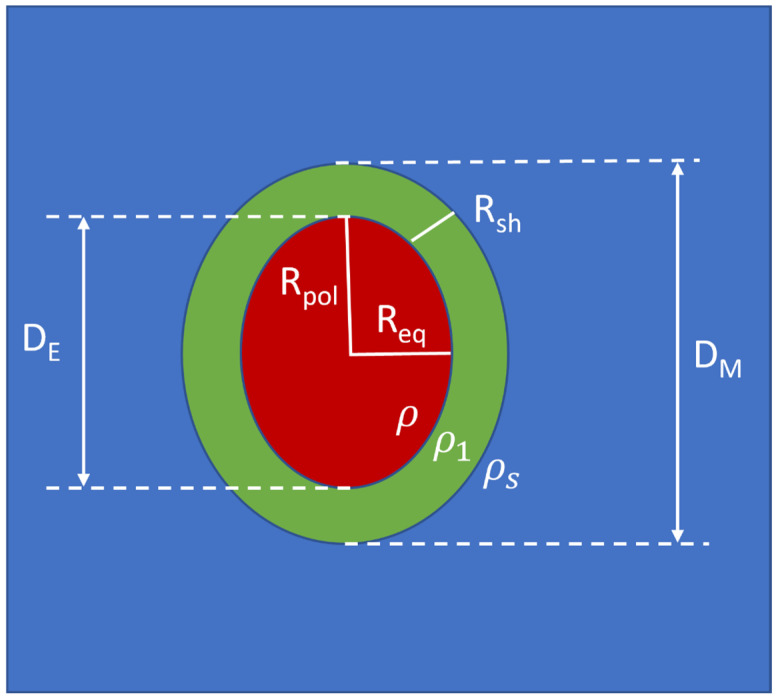
Two-component prolate spheroidal micelles.

Le us assume a core-shell micelle with a spheroidal shape (see Figure 1). To characterize its main structure, we need to determine six unknown quantities: *D_M_*, *R_sh_*, ${\varepsilon ,\;N_{agg},\;\Delta \rho }_{core}=\rho -\rho_{s}$, ${\Delta \rho }_{shell}$ = $\rho_{1}-\rho_{s}$, where (in the schematic model described in Figure 1) *ρ*, $\rho_{1}$ and $\rho_{s}$ are the core, shell, and buffer electron density values, respectively. *D*_M_ and *D*_E_ are the maximum sizes of the whole prolate spheroidal micelle and of its core, respectively, and ε is the ratio of the polar *R_pol_* to the equatorial *R_eq_* core half-sizes (for prolate spheroids, ε > 1; for spheres, ε = 1; for oblate spheroids, ε < 1). For prolate spheroids, *D_M_* coincides with the maximum size of the micelle, namely *D_max_*. For oblate spheroids (ε < 1), *D_M_* = ε*D_max_*.

In this section, we will show that the size of the shell and the whole micelle can be directly obtained by the analysis of the PDF derivative. Indeed, defining γ(*r*) as the autocorrelation function, averaged on the whole solid angle, of the excess/defective scattering density inside the micelle with respect to the surrounding buffer value, we have PDF(*r*) = *r*^2^γ(*r*) [14]. The autocorrelation function of any box function of width *t* is a triangular function of half width *t,* and its first derivative has a distance between maximum and minimum equal to *t*. Therefore, for ideal micelles of spheroidal shape, made of a core of equatorial size *R_eq_* and a shell of size *R_sh_* < *R_eq_*, as schematically shown in Figure 1, due to the properties of the autocorrelation function, the distance between the maximum and the neighborhood minimum of the PDF first derivative should be equal to the minimum size between *R_sh_* and *R_eq_*, i.e., usually *R_sh_* (half width of the autocorrelation function of the shell). However, in real micelles, the region surrounding the hydrophobic core can be viewed as a polymer solution of hydrophilic chains and water (or buffer solution), characterized by the fact that the hydrophilic chains are fixed, at one end, to the hydrophobic core but can move freely at the other end, implying a continuous random chain deformation and a consequent local fluctuation of the shell size and the micelle’s shape [1].

**Figure 2:**
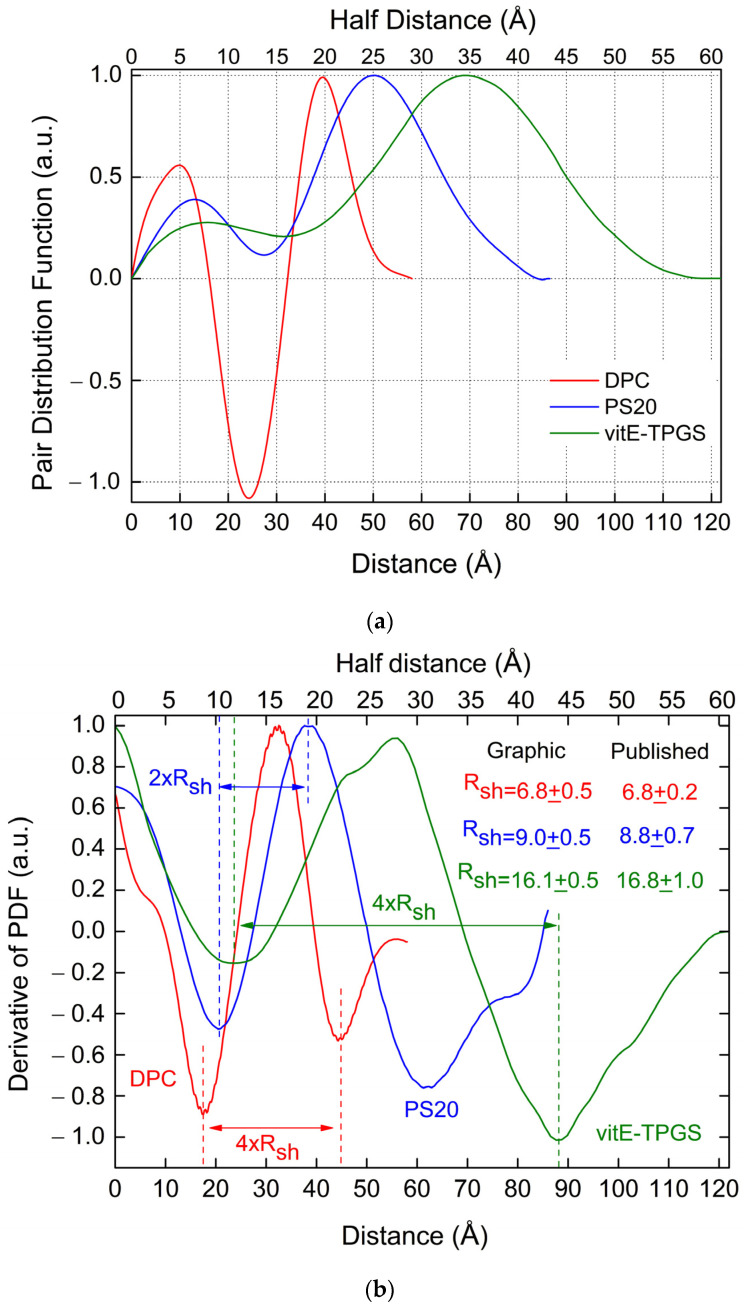
(**a**) PDF(*r*) derived from the SAXS data measured/published on micelles made of three different types of monomers: the blue curve for PS20 [12], the red curve for DPC [13], and the green curve for VitE-TPGS [4]. (**b**) First derivative of the PDF(*r*) reported in (**a**): the blue curve for PS20 [12], the red curve for DPC [13], and the green curve for VitE-TPGS [4].

Figure 2a displays the PDF functions derived from the SAXS data measured/published on micelles made by three different types of monomers: the blue curve for PS20 [12], the red curve for DPC [13], and the green curve for VitE-TPGS [4]. Figure 2b shows the corresponding PDF first derivatives for the same three cases. By comparing the published values of the shell-size with the PDF derivative’s widths, we see that the distance between the maximum and its neighborhood minimum is not equal to *R_sh_* but to 2*R_sh_*. Similarly, the distance between the two minima closer to the maximum is equal to 4*R_sh_* instead of 2*R_sh_*. The values of *R_sh_* obtained either by the distance between the two minima closer to the maximum or by the distance between the maximum and the neighborhood left minimum are quite equivalent if the main peak’s shape is not too asymmetrical with respect to its maximum value. For this reason, the value of *R_sh_* obtained by the distance between the two minima closer to the maximum can be selected as the default. However, for very asymmetric main peaks, it is preferable to select the *R_sh_* value obtained by the distance between the maximum and the left neighborhood minimum.

We compared the values derived graphically by the difference of the maximum and minimum derivative’s distances to the values published in the three cited works [4,12,13], showing a good agreement for the shell size (see the legend of Figure 2b, where the sizes are given in Å).

This double distance between maxima and minima in the PDF derivative can be explained by the fact that the hydrophilic part of the micelle has a variable thickness ranging from 0 to *R_sh_*, caused by random movements of the hydrophilic chains into the buffer liquid. These random variations of the shell thickness influence the actual PDF size values. Indeed, if *x*, *y* are discrete independent random shell size fluctuations along two independent axes of the micelle, with probability functions *f*(*x*) and *g*(*y*), then the probability that *x* + *y* = *d* is the sum of possible values of *x* of the product *f*(*x*) × *g*(*d* − *x*). This is the definition of a convolution, which could be mathematically taken into account in the PDF by considering it convolved with a suitable function of width $\sqrt{1+1}$ × *R_sh_* = $\sqrt{2}$*R_sh_*, the variations of the shell thickness along the two considered axes, being two independent random processes. The same happens for three independent random shell size fluctuations along the three principal axes of the spheroid-shaped micelle, implying a convolution of PDF with a suitable function of width $\sqrt{3}$*R_sh_*. Therefore, the difference between the maximum and minimum of the derivative of the PDF, which is the integral of all angular directions over the whole solid angle, will be characterized by a width of the order of $\sqrt{3+1}$ × *R_sh_* = 2*R_sh_*, i.e., twice the shell size, i.e., twice the value that we would have expected for an ideal shell without any random size variation. This relationship is verified in Figure 2b for all three considered experimental cases [4,12,13], on micelles made by three different monomers. This property can be used for a quick and reliable estimate of the shell size directly from the distances between peaks of the PDF derivative, as previously shown. Moreover, given the maximum distance, *D*_max_, determined from the PDF profile where it falls to 0, the quantity *D*_max_/2 − *R_sh_* can then be readily calculated. This is the polar core half-size R_pol_ for prolate spheroids or the equatorial core half-size R_eq_ for oblate spheroids.

Thus, only four unknown quantities remain to be determined to completely solve the micelle structure: the ellipticity of the shape ε; the aggregation number, i.e., the number of monomers constituting the micelle, $N_{agg}$; the core and shell electron density contrast’s values ${\Delta \rho }_{core}={\Delta \rho }_{E}=\rho -\rho_{s}$, ${\Delta \rho }_{shell}$ = ${\Delta \rho }_{P}=\rho_{1}-\rho_{s}$.

For this further step, we introduce a generalization of the equation that gives the radius of gyration for a particle with an electron density ρ(*r*). Indeed, the gyration radius is a particular case of the following equation, when *n* = 2:

(1) $$R^{n}=\left(\int r^{n}\left(\rho \left(r\right)-\rho_{s}\right)d^{3}r\right)/\left(\int \left(\rho \left(r\right)-\rho_{s}\right)d^{3}r\right).$$

The radius of gyration (*n* = 2 in Equation (1)) is a measure of the atomic electron density within the micelle as a function of the quadratic distance *r*^2^ from its center [4]. Other exponents in Equation (1) would give complementary information, with respect to the gyration radius, about the electron-density distribution inside the micelle, like moments do for a probability distribution.

In Appendix A, we have derived, for *n* = 1, 2, 4, 6, and for a two-component spheroid-shaped micelle, the analytical expression of the above integrals. Comparing the analytical expression of the *n*-th *r*-power obtained by Equation (1) with the corresponding *n*-th *r*-power integral of the PDF

(2) $$R_{PDF}^{n}=\frac{\int_{0}^{D_{max}}PDF\left(r\right)r^{n}dr}{2^{n-1}\int_{0}^{D_{max}}PDF\left(r\right)dr},$$

where $D_{max}$ is the maximum distance in the PDF, we can derive four equations in the four unknows ${\varepsilon ,\;N_{agg},\;\Delta \rho }_{core}={\Delta \rho }_{E}=\rho -\rho_{s}$, ${\Delta \rho }_{shell}$ = ${\Delta \rho }_{P}=\rho_{1}-\rho_{s}$. (Equations (A13), (A22) and (A31)). Equation (2) is analogous to the *n*-th moments of a probability distribution, calculated with respect to a constant value. In the case of Equation (2), this constant value is set to zero, and, thus, for *n* = 1, we obtain the mean (average radius); for *n* = 2, we obtain the variance (gyration radius); and for *n* = 4 and *n* = 6, we obtain, respectively, equivalent of kurtosis and the hypertailedness [15].

Moreover, if the PDF is expressed on an absolute scale, we can derive a further equation [2]:

(3) $$I\left(0\right)=4\pi \int_{0}^{D_{M}}PDF\left(r\right)dr=\frac{K}{N_{agg}}{V_{M}^{2}\left({\Delta \rho }_{P}+\left({\Delta \rho }_{E}-{\Delta \rho }_{P}\right)\frac{V_{E}}{V_{M}}\right)}^{2},$$

where V_M_ and V_E_ are the micelle and core volume, respectively, and K is a quantity related to the concentration of monomers in solution (see Appendix A). Adding this further equation to the previous four, we obtain an overdetermined system of equations in the four unknowns. In turn, this gives us the possibility of defying a Figure of Merit (FOM), described in Appendix A, useful to find the more probable solution of the system of analytical equations. This is fundamental, considering that the analytical equations are polynomial ratios depending on powers of the unknowns, thereby allowing for more than one possible physical solution. The non-linearity of the analytical equations derived by the PDF visualizes in mathematical terms the reason why different software gives different solutions, even starting from the same experimental SAXS dataset.

**Figure 3:**
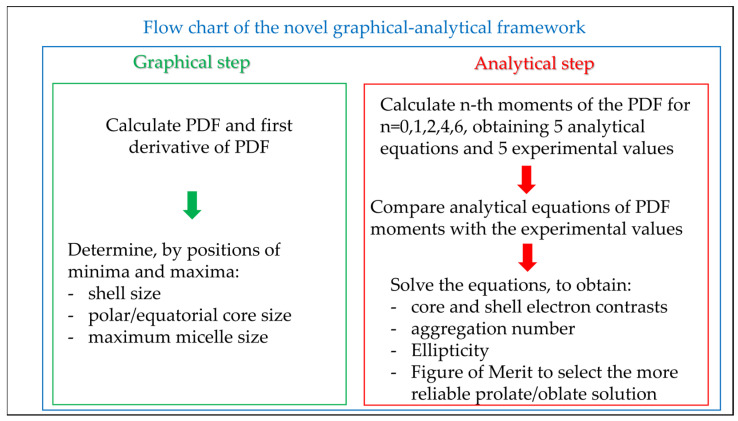
Flow chart of the proposed new method for determining the structural parameters of core-shell spheroidal micelles.

Figure 3 summarizes in a flow chart the new proposed graphical-analytical approach presented here and in detail in Appendix A.

A general question is regarding the maximum number of independent parameters that can in principle be extracted from the SAXS data about micelles’ structure under study. If we assume a spheroidal core-shell micelle, as in this work, we have six free parameters that must be determined: core and shell electron contrasts and sizes, aggregation number, and polar/equatorial asymmetry (ellipticity). A measure of information content in SAXS data is provided by Shannon’s sampling theorem: the value of the minimum scattering vector *s_min_* should not exceed the first Shannon channel (π/*D*_M_), with a total number of channels proportional to *D*_M_ × *s_max_*/π, where *s_max_* is the maximum measured scattering vector not affected by excessive levels of noise [14].

This constraint puts limits on the use of indirect transformation methods used for determining PDF from SAXS data. Usually, existing theoretical approaches either simulate SAXS curves or calculate the PDF profile from SAXS data and compare point-by-point theoretical predictions and experimental results until a minimum difference is reached. However, it is a common experience when using existing software, as described in Section 1, that even small variations in the selected scattering range used to calculate PDF from SAXS data may cause changes in the PDF profiles. The new approach here proposed, based on the theoretical calculation and experimental determination of the *n*-th moments of the PDF, given by Equation (2), should be less affected by small differences in the selected scattering range of SAXS data used for solving the unknown structure. Indeed, moments of a distribution are integral values calculated over the whole range of distances, and, for this reason, they are less sensitive to variations in the PDF shapes related to the selected scattering ranges.

The above graphical-analytical approach, detailed in Appendix A, has been applied in the following sections to several experimental datasets already published in three different works: PS20 [12], DPC, and SDS [13], VitE-TPGS with and without a PSC [4], to confirm the ability of this new approach to provide the correct structural parameters under the assumption of spheroidal core-shell micelles.

## 3. Application of the Graphical-Analytical Approach to Micelles

### 3.1. PS20 Micelles

Table 1 summarizes some properties of PS20. Le Maire et al. [16] evaluated the mass density of PS20 as equal to 1.1507 g/cm^3^ (data reported for 25 °C). The PS20 properties summarized in Table 1 have been derived by using the above mass density’s value.

In [12], the number of monomers aggregated in the micelle *N_agg_* = 34 was estimated as *I*(0)/*I_PS20_*, where *I_PS20_* = 0.227 × 10^−3^ cm^−1^ is the computed forward SAXS scattering by PS20 molecules dispersed in water and *I*(0) is the measured forward SAXS scattering of the entire PS20 micelle, placed on an absolute scale. From Equation (A27), reported in Appendix A, for a unit concentration, we obtain *I_PS20_* = 0.235 × 10^−3^ cm^−1^/unit concentration, very close to the value reported in [11], but a quite different value of *N_agg_*. Indeed, from [12], we have: *I*(0) = 0.054 cm^−1^; $c_{mon}$ = 5 mg/mL = 0.005 g/cm^3^; cmc = 0.06 mg/mL (0.049 mM), $K=\frac{\left(c_{mon}-cmc\right)N_{A}{r_{e}}^{2}}{{MW}_{mon}}=1.93\times 10^{-7}\;{cm}^{-1}{n_{e}}^{-2},$ $r_{g}=34.0\pm 1.0\;Å$. The *I*(0) value, normalized per unit concentration, is 0.0108 cm^−1^/unit concentration. This value divided by *I_PS20_* = 0.227 × 10^−3^ cm^−1^/unit concentration gives *N_agg_* ~ 48, and not 34 as indicated in [12]. In any case, by using *I_PS20_* = 0.235 × 10^−3^ cm^−1^/unit concentration, here derived, we obtain *N_agg,ini_* = 46. Alternatively, as a second estimate of *N_agg_*, inserting the length of the alkyl chain (lauric acid), made of 12 carbon atoms, constituting the PS20 monomers, into Equations (A24) and (A25) of Appendix A, we obtain *N_agg,ini_* = 55. Averaging these two values (*N_agg,ini_* = 46 and *N_agg,ini_* = 55), we have *N_agg,ini_* = 50 ± 5. This is the first estimate of the number of monomers constituting the micelles, which is inserted in Equation (A22) of Appendix A for calculating the *N*_agg_ derived by solving the analytical equations.

Table 2 summarizes the core and shell sizes obtained for PS20 micelles [12] versus the values here derived from our graphical approach, described in the previous section, showing quite good agreement.

**Figure 4:**
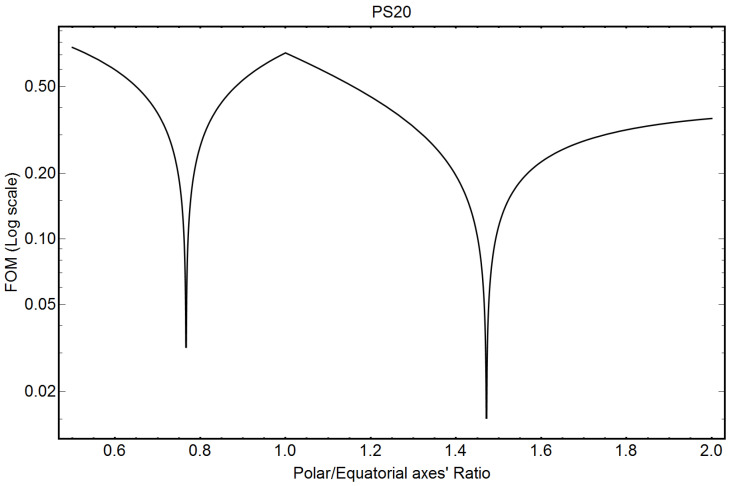
Figure of Merit (FOM) (Equation (A33) of Appendix A) for PS20 micelles. The minimum of the FOM indicates the solution.

Figure 4 shows the FOM output for PS20, as found by Equation (A31) of Appendix A. The minimum of the FOM indicates a prolate shape as the most reliable solution, with ellipticity values very close to those published in [12].

Table 3 compares the ellipticity factor, the electron density contrast’s values and the number of monomers constituting the micelles, published in [12], with the corresponding values obtained by our analytical formulae.

Within experimental errors, a good agreement is obtained between the analytical results here obtained and the results published in [12]. The higher electron density contrast values reported in [12], with respect to those here derived, can be attributed to the fact that, from Table 2, the value of the maximum core ellipsoid semiaxis, *D_M_*/2 − *R_sh_*, published in [12], seems to be overestimated and not compatible with the maximum size of the micelles (*D_M_* = 86.0 Å), as evidenced in the note of Table 2.

### 3.2. DPC Micelles

Table 4 summarizes the chemical/physical properties of DPC, obtained assuming a mass density close to 1 g/cm^3^.

All the experimental data reported in the following, regarding the DPC micelles, have been taken from [13]. Table 5 summarizes the core and shell sizes obtained for DPC micelles [13] versus the values here derived from the graphical approach (derivative of the PDF).

An excellent agreement is obtained. From ref. [13], for DPC, we have: *I*(0) = 0.0047 cm^−1^; $c_{mon}$ = 5 mg/mL; cmc = 0.31 mg/mL, $K=\frac{\left(c_{mon}-cmc\right)N_{A}{r_{e}}^{2}}{{MW}_{mon}}=6.4\times 10^{-7}\;{cm}^{-1}{n_{e}}^{-2}$. Moreover, in [13], *N_agg_* = 56 is obtained by the fitting model (Table 2 of [13]). Inserting the length of the alkyl chain made of 12 carbon atoms, constituting the DPC monomers, into Equations (A24) and (A25) of Appendix A, we obtain *N_agg,ini_* = 55. The gyration radius can be computed by the PDF by means of Equation (A11), obtaining $r_{g}=32.1\pm 0.1$ Å, a value very different from the published value of 37.5±2.0 Å, derived by the low-angle SAXS intensity’s analysis (Guinier approximation), also called “reciprocal space” gyration radius [17]. The PDF-derived gyration radius, also called “real-space” gyration radius, given by Equation (A11), has the advantage of being derived from the entire scattering curve and not just the lowest-resolution data [17]. Therefore, it is more representative of the atoms’ distribution within the micelle. The electron density values are strongly affected by the value of the gyration ratio if the low-resolution (reciprocal-space) estimation is too different from the real-space value.

**Figure 5:**
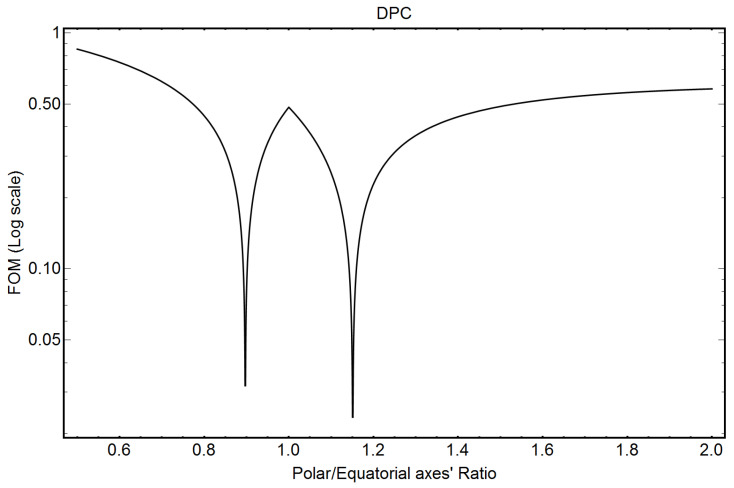
Figure of Merit (FOM) (Equation (A33) of Appendix A) for DPC micelles. The minima of the FOM indicate possible solutions.

Figure 5 shows the FOM output for DPC (Equation (A31)) of Appendix A. The two minima of the FOM indicate possible solutions. The FOM indicates almost equivalent prolate and oblate-shaped solutions, not far from the spherical shape.

To discriminate between the two possible solutions, namely $\varepsilon_{P}$ and $\varepsilon_{O}$, for the prolate and oblate shapes, respectively, we can define a relative probability P[ε] associated with the precision with which Equations (A21) and (A30), reported in Appendix A, are satisfied:

(4) $$P\left[\varepsilon_{P,O}\right]=1-\frac{FOM[\varepsilon_{P,O}]}{FOM[\varepsilon_{P}]+FOM[\varepsilon_{O}]}.$$

Equation (4) gives 0.61 and 0.39 as relative probabilities for the prolate and oblate solutions, respectively, indicating that the oblate shape could also be actually competitive with respect to the prolate one. As a comparison, the oblate solution for PS20 (see Figure 4) has a relative probability of only 0.14, which is very low with respect to the prolate one (1 − 0.14 = 0.86).

Table 6 compares the ellipticity factor, the electron density contrast’s values, and the number of monomers constituting the micelles, published in [13], with the corresponding values obtained by our analytical formulae. Let us note that the core electron density contrast published in [13], ${\Delta \rho }_{E}$ = −0.066±0.003 *n_e_*/Å^3^ is 80% larger than that of lipid tails (−0.036 *n_e_*/Å^3^) [12], leading to a too low electron density of the core: 0.268 *n_e_*/Å^3^.

Moreover, in [11], small-angle neutron scattering was used to investigate the size and shape of DPC micelles. The authors of this study, at a $c_{mon}$ = 100 mM, have obtained *R_pol_* = 20.2 ± 0.5 Å, *R_sh_* = 6.9 ± 2.0 Å, in good agreement with all the values in Table 5. In addition, in [11], the authors determined an ellipticity value of ε = 1.22 ± 0.07, which is in good agreement with the ellipticity value here derived (1.15 ± 0.07), confirming that ε = 1.52 ± 0.014, reported in [13], is too large, probably caused by the overestimation of the gyration radius previously described.

### 3.3. VitE-TPGS Micelles

Table 7 summarizes the physical properties of VitE-TPGS. 

From our graphical analysis of the PDF derivative, we have found *R_sh_* = 16.1 ± 0.5 Å, in agreement with the value reported for sample S5 in [4], and *D_M_* = 121.0 ± 0.5 Å. In [4], we have assumed a spherical shape for the VitE-TPGS micelles and convolved the PDF theoretical predictions, given the model, with a gaussian function of width *σ* = 28 ± 0.5 Å to describe non-ideal core-shell and shell-buffer interfaces. In the comparison of the results published in [4] with those here obtained, we also need to consider the widening of the PDF profiles due to this convolution. Table 8 summarizes the core and shell sizes obtained for VitE-TPGS micelles [4] versus the values here derived from the graphical approach (derivative of the PDF).

Even if the values published in [4] have been obtained under the assumption of a spherical shape, from Table 8, we note a good agreement between the results obtained by the two different approaches within the errors’ bars.

**Figure 6:**
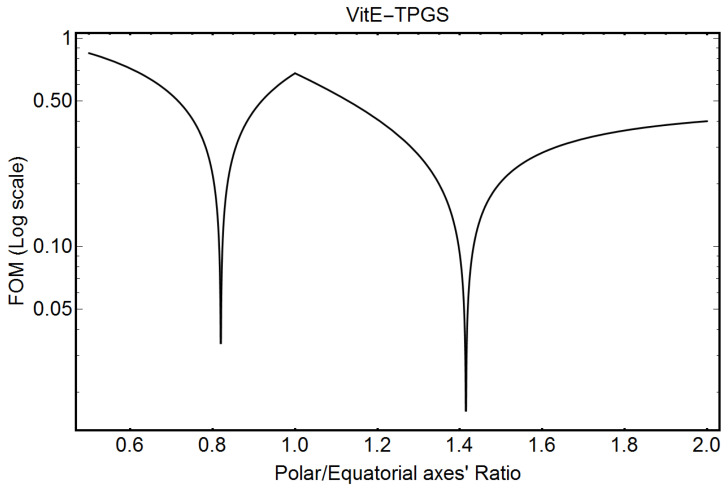
Figure of Merit (FOM) (Equation (A33) of Appendix A) for VitE-TPGS micelles. The minimum of the FOM indicates the solution.

Figure 6 shows the FOM output for VitE-TPGS micelles evaluated by Equation (A31) of Appendix A. The minimum of the FOM indicates the solution. Equation (4) gives 0.81 and 0.19 as relative probabilities for the prolate and oblate solutions in Figure 6, respectively.

Table 9 compares the ellipticity factor, the electron density contrast’s values, and the number of monomers constituting the VitE-TPGS micelles, published in [4], with the corresponding values obtained by the analytical formulae here derived.

From Table 9, we note still a good agreement between the results of the two different approaches, despite the different shapes, i.e., sphere versus spheroid, assumed in the analysis of the PDF data. Indeed, the differences in the values of electron density contrasts of the prolate ellipsoid with respect to the sphere are within 2 standard deviations.

### 3.4. VitE-TPGS Micelles with Eltrombopag (PSC)

**Figure 7:**
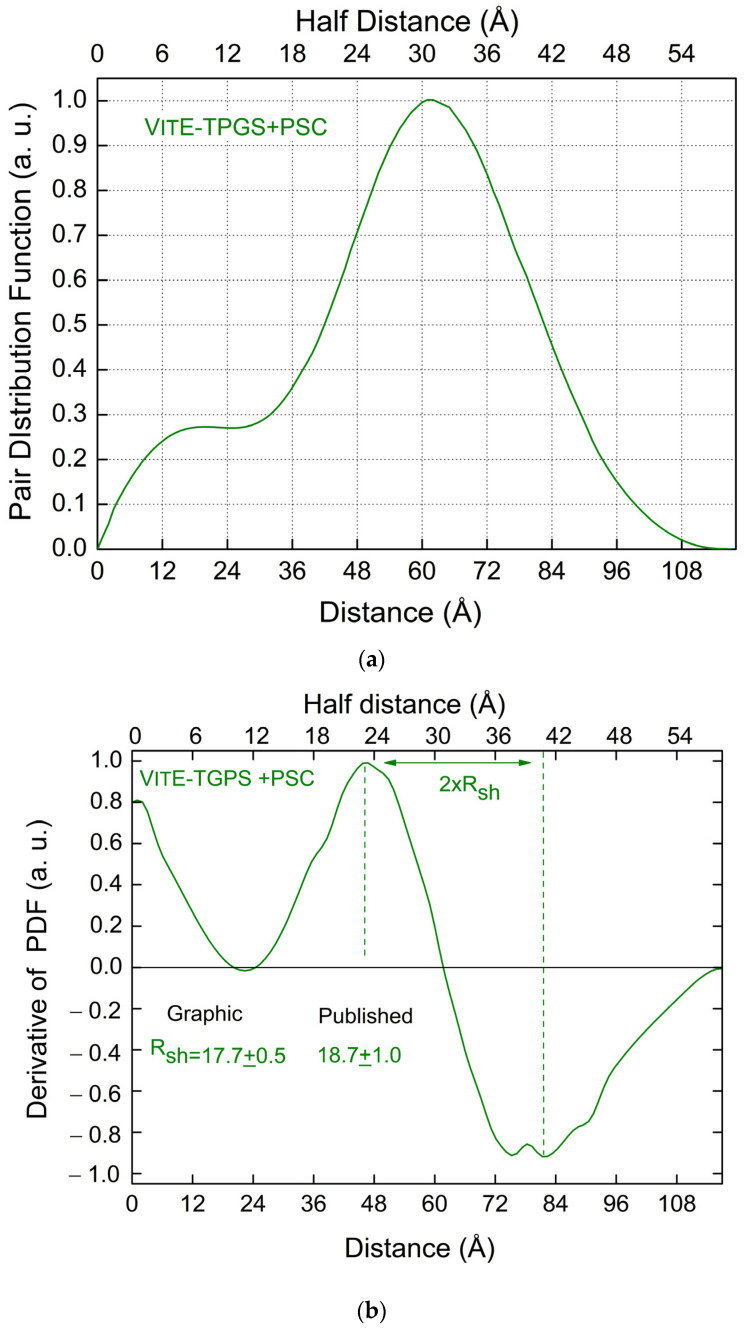
(**a**) PDI derived from the SAXS data measured on micelles made by VitE-TPGS with Eltrombopag [4]. (**b**) First derivative of the PDF reported in (**a**) (VitE-TPGS with Eltrombopag).

Figure 7a shows the PDF function derived from the SAXS data measured on micelles made by VitE-TPGS with Eltrombopag [4]. Figure 7b shows the corresponding PDF first derivative.

It is interesting to note the presence of two very close minima in Figure 7b in the region between 72 and 84 Å, a feature that is not visible in the green curve of Figure 2b, obtained for VitE-TPGS micelles without PSC. This feature can be explained by the presence of PSC molecules (Eltrombopag) linked to the VitE-TPGS hydrophobic-hydrophilic interface in the region of the linker, as discussed in [4]. It is worth noting that the first derivative of the PDF seems to provide a direct visualization of a change in the structure of the micells after the drug loading, as discussed in [4].

From our graphical analysis of the PDF derivative, we find *R_sh_* = 17.7 ± 0.5 Å, in agreement with the value reported for sample S7 in [4], and *D_M_* = 117.0 ± 0.5 Å. In [4], we have assumed a spherical shape for the VitE-TPGS micelles and convolved the PDF theoretical predictions, given the model, with a gaussian function with a width *σ* = 29 ± 0.5 Å to describe non-ideal core-shell and shell-buffer interfaces. By comparing the results published in [4] with those here obtained, we also need to consider the widening of the PDF profiles due to this convolution. Table 10 summarizes the core and shell sizes obtained for VitE-TPGS micelles with PSC [4] versus the values derived here from the graphical approach (derivative of the PDF).

Even if the values published in [4] had been obtained under the assumption of a spherical shape, from Table 10, we note a good agreement between the results obtained by the two different approaches within the errors’ bars. 

Table 11 compares the ellipticity factor, the electron density contrast’s values, and the number of monomers constituting the (VitE-TPGS plus PSC) micelles, published in [4], with the corresponding values obtained by the analytical formulae derived here.

From Table 11, even in the case of PSC-loaded micelles, we note a good agreement between the results of the two different approaches, despite the different shape, i.e., sphere versus spheroid, assumed in the analysis of the PDF data. The value of the ellipticity confirms the prolate shape, even if the asymmetry between the polar and equatorial sizes is slightly larger for the PSC-loaded micelles with respect to the unloaded PSC case discussed in the previous sub-section.

### 3.5. SDS Micelles

Table 12 summarizes the chemical/physical properties of SDS, obtained for a mass density of 1.1 g/cm^3^.

**Figure 8:**
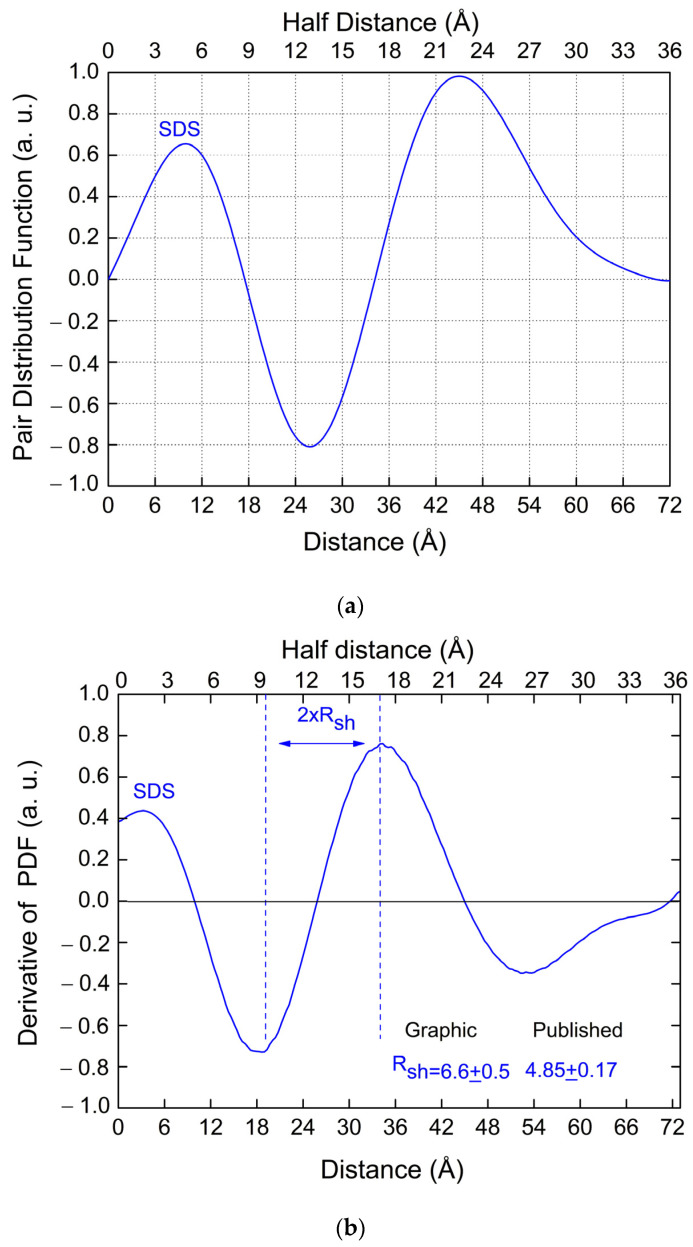
(**a**) PDF derived from the SAXS data for SDS micelles published in [13]. (**b**) First derivative of the PDF reported in (**a**) (SDS micelles).

Figure 8a shows the PDF function for SDS micelles published in [13]. Figure 8b shows the corresponding PDF first derivative.

All the experimental data reported in the following, regarding the SDS micelles, have been taken from [13]. Table 13 summarizes the core and shell sizes obtained for SDS micelles [13] versus the values here derived from the graphical approach (derivative of the PDF).

A good agreement is obtained between the results obtained from our model and the previously published data. From ref. [13], for SDS, we have: *I*(0) = 0.01875 cm^−1^; $c_{mon}$ = 6.25 mg/mL; cmc = 0.21 mg/mL, $K=\frac{\left(c_{mon}-cmc\right)N_{A}{r_{e}}^{2}}{{MW}_{mon}}=4.2\times 10^{-7}\;{cm}^{-1}{n_{e}}^{-2}$. Moreover, in [13], *N_agg_* = 90 has been obtained from the *I*(*0*) value and *N_agg_* = 118 from the model (Table 2 of [13]). The gyration radius can be computed by the PDF by means of Equation (A11), obtaining $r_{g}=33.0\pm 0.1$ Å, which coincides with the value reported in [13].

Table 14 compares the ellipticity factor, the electron density contrast’s values, and the number of monomers constituting the micelles, published in [13], with the corresponding values obtained by our analytical formulae. Within the experimental errors, a good agreement is again obtained.

## 4. Conclusions and Perspectives

In this study, we present an innovative theoretical framework designed for analyzing Pair Distribution Function (PDF) data derived from Small Angle X-ray Scattering (SAXS) measurements of core-shell micelles. Our approach leverages the first derivative of the PDF and employs novel analytical equations to determine key structural parameters: the sizes of the core and shell, the number of monomers constituting the micelles, the degree of ellipticity, and the contrast in electron density between the core-shell and shell-buffer regions. The analysis is conducted under the hypothesis of spheroid-shaped, two-component micelles.

The results derived from our innovative approach for four distinct surfactants, including, for one of them, micelles with and without a PSC, exhibit strong concordance with previously published data, obtained through diverse data analysis methodologies. This demonstrates the validity and robustness of our method for spheroidal micelles.

The significance of this innovative approach lies in its versatility and simplicity, enabling rapid implementation of the analytical formulae. Calculation of derivatives and generating outputs from the analytical formulae can be readily achieved using widely accessible software for data analysis. There is no requirement for sophisticated tools to simulate SAXS and PDF data. Our proposed graphical-analytical approach is equally well-suited for analyzing both synchrotron and laboratory bio-SAXS data and delivers accurate and insightful results, provided optimal background correction is performed and scattered intensity is expressed on an absolute scale.

This novel approach could become an invaluable tool in the context of discovering, developing, and characterizing new surfactants for pharmaceutical applications. It enables systematic investigations of micelle characteristics and behaviors across a set of various experimental conditions, such as temperature, mechanical stress, pH, active ingredient concentration, and storage container, facilitating the identification of optimal conditions. Additionally, it can be a powerful tool to support the screening of large libraries of new micelles generated from multiple combinations of hydrophilic, linker, and hydrophobic compounds.

Every theoretical approach has its own limits of application. In fact, SAXS curves decay rapidly with the scattering vector. The maximum value is usually of the order of 2–3 nm^−1^, so the total number of Shannon channels is typically of the order of 5–10. In turn, this finding limits the number of free parameters of the micelles’ structure under study that can be readily determined by SAXS data. The spheroidal core-shell model presented here, with six free parameters, is a good compromise to handle the above-described limits, providing already several structural details that are very useful for studying new surfactants for pharmaceutical applications.

In principle, this novel analytical framework might potentially be extended to other micelle shapes, but this effort would require some further studies. In any case, as the examples in Section 3.3 and Section 3.4 have shown, changing the assumed shape from spherical to spheroidal causes only small variations in the obtained electron density contrasts. The reason is related to the fact that in SAXS experiments we measure solid-angle averaged values of the shape. Therefore, the correct quantitative scale of electron density contrasts can still be evaluated, assuming a shape that only approximates the actual one.

Similarly, this analytical framework might be applicable to some other nanoparticles, such as aggregated proteins, viruses, etc., provided that, at the resolution scale approached by scattering experiments, their actual shape can be reasonably well approximated with a two-component spheroidal shape. In this regard, as discussed in Section 2, we should also take into account that the minimum scattering vector should not exceed the first Shannon channel (π/*D_M_*). If the size of the scattering nano-objects increases, the first Shannon channel’s constraints become more stringent, limiting the maximum nano-object size to a few tens of nanometers. However, collecting SAXS data at dedicated beamlines where sample-to-detector distances can reach several tens of meters may permit us to overcome this limitation.

Drug-loaded micelles, as discussed in Section 3, could present a spheroidal internal structure more complex than a two-component case, here assumed. In that case, it will be necessary to assume a micelle structure consisting of more than two components. The inspection of the first derivative of the PDF, here proposed, could give some useful information about the presence of more than two components in the structure of the studied micelles, and the model could then be extended with an additional shell, as already discussed in [4], to have more detailed information on the influence of the loaded drug on the micelles’ shape as well as on the spatial position of the drug, either inside the micelle or linked to its hydrophobic-hydrophilic interface, as demonstrated for vitE-TPGS added with PSC [4].

## Figures and Tables

**Table 1:** Physical/chemical properties of the PS20 surfactant. *N_A_* is the Avogadro number. Mass density = 1.1507 g/cm^3^ [16].

Compound	Mole Mass (g)	*N_e_* = *n_e_*/Molecule	Monomer or Molecule Volume (Å^3^)	Mole/L	*n_e_*/L	Electron Density ρ (*n_e_*/Å^3^)
PS20 C_58_H_114_O_26_	1227.54	670	1771	0.938	616.4 × *N_A_*	0.378
Water H_2_O	18.016	10	29.9	55.51	555.1 × *N_A_*	0.334

**Table 2:** Core (*D_M_*/2 − *R_sh_*) half-size, shell (*R_sh_*) half-size, and maximum micelle size (*D_M_*) obtained by the graphical analysis of the PDF of PS20 micelles versus the values published in [12]. Concentration of the monomer: 5 mg/mL. The last column indicates the maximum core ellipsoid semiaxis, *D_M_*/2 − *R_sh_*, which is the polar core half-size *R_pol_* for prolate spheroids and the equatorial core half-size *R_eq_* for oblate spheroids.

Method	*R_sh_* (Å)	*D_M_* (Å)	*D_M_*/2 − *R_sh_* (Å)
Published [12]	8.8 ± 0.7	86.0 ± 0.5	36.8 ± 0.7 (*)
Graphical	9.0 ± 0.5	86.0 ± 0.5	34.1 ± 0.5

(*) In the table, we have reported the published value of 36.8 Å, although a check gives 2 × 36.8 + 2 × 8.8 = 91.2 ± 2.8 Å, a value too large compared with the maximum size of the micelle, *D*_M_ = 86 Å. From the maximum micelle size and shell size published in [12], reported in the first two columns, we should expect *D_M_*/2 − *R_sh_* = 34.2 Å as the value of the third column, and not 36.8.

**Table 3:** Summary of the ellipticity, aggregation number, and the core and shell electron density contrasts’ values obtained in [12] versus the values here derived by the analytical formulae.

Method	ε	*N_agg_*	$\Delta \rho_{E}$ (*n_e_*/Å^3^)	${\Delta \rho }_{P}$ (*n_e_*/Å^3^)
Published [12]	1.50 ± 0.06	34	−0.035 ± 0.002	0.060 ± 0.004
Published [12]	1.50 ± 0.06	34	−0.031 ± 0.002	0.064 ± 0.003
Analytical	1.47 ± 0.01	35 ± 1	−0.030 ± 0.002	0.050 ± 0.005

**Table 4:** Summary of the chemical/physical properties of the dodecyl phosphocholine (DPC) experiment. *N_A_* is the Avogadro number. Assumed mass density of 1 g/cm^3^.

Compound	Mole Mass (g)	*N_e_* = *n_e_*/Molecule	Monomer or Molecule Volume (Å^3^)	Mole/L	*n_e_*/L	Electron Density ρ (*n_e_*/Å^3^)
DPC C_17_H_38_NO_4_P	351.5	194	548	3.03	587.9 × *N_A_*	0.354
H_2_O	18.016	10	29.9	55.51	555.1 × *N_A_*	0.334

**Table 5:** Core (*D_M_*/2 − *R_sh_*) half-size, shell (*R_sh_*) half-size, and maximum micelle size (*D_M_*) obtained by the graphical analysis of the PDF of DPC micelles compared with the values published in [13]. Concentration of the monomer: 5 mg/mL. The last column indicates *D_M_*/2 − *R_sh_*, which is the polar core half-size *R_pol_* for prolate spheroids the equatorial core half-size R_eq_ for oblate spheroids.

Method	*R*_sh_ (Å)	*D*_M_ (Å)	*D*_M_/2 − *R*_sh_ (Å)
Published [13]	6.83 ± 0.22	58.1 ± 1.2	22.21 ± 0.38
Graphical	6.8 ± 0.5	58.0 ± 0.5	22.2 ± 0.5

**Table 6:** Summary of the ellipticity, aggregation number, and the core and shell electron density contrasts’ values obtained in [13] versus the values here derived by the analytical formulae for prolate and oblate shapes.

Method	ε	*N*_agg_	${\Delta \rho }_{E}$ (*n_e_*/Å^3^)	${\Delta \rho }_{P}$ (*n_e_*/Å^3^)
Published [13]	1.52 ± 0.014	56	−0.066 ± 0.003	0.054 ± 0.004
Analytical	1.15 ± 0.07	57	−0.043 ± 0.003	0.041 ± 0.007
Analytical	0.90 ± 0.07	57	−0.041 ± 0.003	0.038 ± 0.007

**Table 7:** Summary of the VitE-TPGS physical properties. *N_A_* is the Avogadro number. Mass density of 1.08 g/cm^3^ [4].

Compound	Mole Mass (g)	*N_e_* = *n_e_*/Molecule	Monomer or Molecule Volume (Å^3^)	Mole/L	*n_e_*/L	Electron Density ρ (*n_e_*/Å^3^)
vitE-TPGS C_33_O_5_H_54_(CH_2_CH_2_O)*_n_* *n* = 0.7 × 22 + 0.3 × 23	1513.1	827	2327	0.716	592.1 × *N_A_*	0.357
H_2_O	18.016	10	29.9	55.51	555.1 × *N_A_*	0.334

**Table 8:** Core (*D_M_*/2 − *R_sh_*) half-size, shell (*R_sh_*) half-size, and maximum micelle size (*D_M_*) obtained by the graphical analysis of the PDF of VitE-TPGS micelles versus the values published in [4]. Concentration of the monomer: 4.1 mg/mL. The last column indicates *D_E_* = *D_M_* − 2*R_sh_*, which is the polar core size for prolate spheroids and the equatorial core size for oblate spheroids.

Method	*R_sh_* (Å)	*σ* (Å)	*D_M_ + σ* (*) (Å)	*D_M_* − 2*R_sh_ + σ* (*) (Å)
Published [4]	16.8 ± 1.0	28.0 ± 0.5	119.5 ± 1.0	85.9 ± 2.5
Graphical	16.1 ± 0.5	0	121.0 ± 1.0	88.8 ± 1.5

(*) In [4], it was assumed to have a spherical shape, and the theoretical results have been convoluted with a gaussian function of width *σ*. Nevertheless, within the experimental errors, we can note a good agreement for the *R*_sh_ derived by the two different approaches. However, for the convolution theorem, we must compare *D_E_* and *D_M_* here, derived with *D_E_* + σ and *D_M_* + σ determined in [4].

**Table 9:** Summary of the structural values obtained for VitE-TPGS micelles in sample S5 of ref. [4] versus the analytical formulae’s values here obtained.

Method	$\Delta \rho_{E}$ (*n_e_*/Å^3^)	${\Delta \rho }_{P}$ (*n_e_*/Å^3^)	ε	$N_{agg}$
Published [4]	−0.037 ± 0.001	0.037 ± 0.001	1 (assumed)	116 ± 1
Analytical	−0.029 ± 0.004	0.033 ± 0.002	1.41 ± 0.002	125 ± 1

**Table 10:** Core (*D_M_*/2 − *R_sh_*) half-size, shell (*R_sh_*) half-size, and maximum micelle size (*D_M_*) obtained by the graphical analysis of the PDF of (VitE-TPGS with PSC)-micelles versus the values published in [4]. Concentration of the monomer: 4.1 mg/mL. The last column indicates *D_E_* = *D_M_* − 2*R_sh_*, which is the polar core size for prolate spheroids and the equatorial core size for oblate spheroids.

Method	*R_sh_* (Å)	*σ* (Å)	*D_M_ + σ* (*) (Å)	*D_M_* − 2*R_sh_ + σ* (*) (Å)
Published [4]	18.7 ± 1.0	29.0 ± 0.5	116.5 ± 1.0	79.1 ± 2.5
Graphical	17.7 ± 0.5	0	116.9 ± 1.0	81.5 ± 1.5

(*) In [4], a spherical shape had been assumed, and the theoretical results had been convoluted with a gaussian function with a width *σ*. Nevertheless, within the experimental errors, we can note a good agreement for the *R_sh_* derived by the two different approaches. However, for the convolution theorem, we must compare *D_E_* and *D_M_* derived here with *D_E_* + σ and *D_M_* + σ determined in [4].

**Table 11:** Summary of the structural values obtained for (VitE-TPGS with PSC) micelles of sample S7 of ref. [4] versus the analytical formulae’s values obtained here.

Method	$\Delta \rho_{E}$ (*n_e_*/Å^3^)	${\Delta \rho }_{P}$ (*n_e_*/Å^3^)	ε	$N_{agg}$
Published [4]	−0.055 ± 0.001	0.045 ± 0.001	1 (assumed)	117 ± 1
Analytical	−0.046 ± 0.002	0.043 ± 0.002	1.45 ± 0.001	123 ± 1

**Table 12:** Summary of the chemical/physical properties of the sodium dodecyl sulfate (SDS) experiment. *N_A_* is the Avogadro number. Mass density of 1.1 g/cm^3^ (https://pubchem.ncbi.nlm.nih.gov/compound/Sodium-dodecyl-sulfate, accessed on 20 April 2024).

Compound	Mole Mass (g)	*N_e_* = *n_e_*/Molecule	Monomer or Molecule Volume (Å^3^)	Mole/L	*n_e_*/L	Electron Density ρ (*n_e_*/Å^3^)
SDS C_12_H_25_SO_4_Na	288.4	156	435.4	3.81	595.0 × *N_A_*	0.358
H_2_O	18.016	10	29.9	55.51	555.1 × *N_A_*	0.334

**Table 13:** Core (*D_M_*/2 − *R_sh_*) half-size, shell (*R_sh_*) half-size, and maximum micelle size (*D_M_*) obtained by the graphical analysis of the PDF of SDS micelles compared with the values published in [13]. Concentration of the monomer: 6.25 mg/mL. The last column indicates *D_M_*/2 − *R_sh_*, which is the polar core half-size *R_pol_* for prolate spheroids or the equatorial core half-size *R_eq_* for oblate spheroids.

Method	*R*_sh_ (Å)	*D*_M_ (Å)	*D*_M_/2 − *R*_sh_ (Å)
Published [13]	4.85 ± 0.17	72.07 ± 4.32	31.185 ± 1.99
Graphical	6.6 ± 0.5	73.0 ± 0.5	29.9 ± 0.5

**Table 14:** Summary of the ellipticity, aggregation number, and core and shell electron density contrasts’ values obtained in [13] for SDS micelles versus the values derived here by the analytical formulae.

Method	ε	*N_agg_*	$\Delta \rho_{E}$ (*n_e_*/Å^3^)	${\Delta \rho }_{P}$ (*n_e_*/Å^3^)
Published [13]	1.75 ± 0.11	90, 118	−0.073 ± 0.006	0.138 ± 0.004
Analytical	1.60 ± 0.07	96 ± 1	−0.082 ± 0.010	0.105 ± 0.003

## Data Availability

No new data were created or analyzed in this study. Data sharing is not applicable to this article.

## Abbreviations

CMC	Critical Micelle Concentration
DPC	Dodecyl phosphocholine
PDF	Pair Distribution Function
PEG	Polyethylene Glycol
PSC	Poorly Soluble Compound
PS20	Polysorbate 20
SAXS	Small-Angle X-ray Scattering
SDS	Sodium Dodecyl Sulfate
VitE-TPGS	D-α-tocopherol polyethylene glycol 1000 succinate

## Appendix A. Derivation of the Analytic Formulae

For two-component micelles with a prolate or oblate spheroid shape (ellipsoids of revolution around the polar axis), the electron density’s contrasts can be defined through the following equations, where subscripts “*E*” and “*P*” refer to core and shell regions, and ρ, $\rho_{1}$, and $\rho_{s}$ are the core, the shell, and the buffer electron density, respectively (see Figure 1 of the main section for the prolate shape):

(A1) $${\Delta \rho }_{E}={\rho -\rho }_{s},\;{{\Delta \rho }_{P}=\rho }_{1}{-\rho }_{s}.$$

The volumes of the whole micelle and of the core can be defined as follows:

(A2) $$V_{M}=\frac{4\pi }{3}\frac{D_{M}}{2}{\left(\frac{D_{E}}{2\varepsilon }+R_{sh}\right)}^{2},\;V_{E}=\frac{4\pi }{3\varepsilon^{2}}{\left(\frac{D_{E}}{2}\right)}^{3},$$

where the maximum sizes of the whole micelle and its core are given by *D_M_* and *D_E_*, respectively, and are schematically shown in Figure 1. Here, ε = *R_pol_*/*R_eq_* is the ratio between the polar *R_pol_* and equatorial *R_eq_* radius of the micelle’s core, and *D_M_* = 2*R_pol_* + 2*R_sh_*, D*_E_* = 2*R_pol_*, where *R_sh_* is the shell size. For prolate spheroids (ε > 1), *D_M_* coincides with the maximum size of the micelle, namely *D_max_*. For oblate spheroids (ε < 1), *D_M_* = ε*D_max_*.

If we calculate the PDF on an absolute scale [cm^−1^/distance], its integral for a two-component spherical micelle can be expressed as follows [2]:

(A3) $$I\left(0\right)=4\pi \int_{0}^{D_{M}}PDF\left(r\right)dr=\frac{K}{N_{agg}}{V_{M}^{2}\left({\Delta \rho }_{P}+\left({\Delta \rho }_{E}-{\Delta \rho }_{P}\right)\frac{V_{E}}{V_{M}}\right)}^{2},$$

where $V_{E}$, $V_{M}$, $\Delta_{E}$ and $\Delta_{P}$ are given by Equations (A1) and (A2). Here,

(A4) $$K=\frac{\left(c_{mon}-cmc\right)N_{A}{r_{e}}^{2}}{{MW}_{mon}},$$

where the surfactant concentration $c_{mon}$ has been corrected by the critical micelle concentration *cmc* expressed in $g/{cm}^{3}$, $N_{A}$ = $6.02214\cdot 10^{23}/mol$ is the Avogadro number, $r_{e}$ is the classic radius of an electron $(2.81794\cdot 10^{-13}cm/e)$, ${MW}_{mon}$ is the molar mass of one monomer, and $N_{agg}$ is the number of aggregated monomers within a micelle.

Moreover, for a two-component spheroid, starting from Equation (SM26) of the Supporting Materials of ref. [4], after some simple algebra, it is possible to show that the gyration radius can be expressed as

(A5) $$R^{2}=\frac{1}{20\varepsilon^{2}}\times \frac{{\Delta \rho }_{P}{X^{2}\left(\varepsilon^{2}+2X^{2}\right)D}_{M}^{5}+\left({\Delta \rho }_{E}-{\Delta \rho }_{P}\right)\left({2+\varepsilon }^{2}\right){\left(D_{M}-2R_{sh}\right)}^{5}}{{\Delta \rho }_{P}{X^{2}D}_{M}^{3}+\left({\Delta \rho }_{E}-{\Delta \rho }_{P}\right){\left(D_{M}-2R_{sh}\right)}^{3}},$$

where

(A6) $$X=1+\frac{2\left(\varepsilon -1\right)R_{sh}}{D_{M}}.$$

Equations (A5) and (A6) are the generalizations of Equation (SM29) of ref. [4], which was obtained under the hypothesis of a spherical shape, to the more general case of an elliptical spheroid.

By using Equations (A3) and (A5) solving for the electron density contrasts, after some algebra, we obtain:

(A7) $${\Delta \rho }_{P}=\frac{6\varepsilon^{2}}{\pi }\sqrt{\frac{N_{agg}I\left(0\right)}{K}}\frac{1}{{X^{2}D}_{M}^{3}}\left(\frac{20\varepsilon^{2}R^{2}-\left({2+\varepsilon }^{2}\right){\left(D_{M}-2R_{sh}\right)}^{2}}{\left(\varepsilon^{2}+2X^{2}\right)D_{M}^{2}-\left({2+\varepsilon }^{2}\right){\left(D_{M}-2R_{sh}\right)}^{2}}\right),\;{\Delta \rho }_{E}={\Delta \rho }_{P}\left[1+\frac{{X^{2}D}_{M}^{3}}{{\left(D_{M}-2R_{sh}\right)}^{3}}\left(\frac{\left(\varepsilon^{2}+2X^{2}\right)D_{M}^{2}-20\varepsilon^{2}R^{2}}{20\varepsilon^{2}R^{2}-\left({2+\varepsilon }^{2}\right){\left(D_{M}-2R_{sh}\right)}^{2}}\right)\right]$$

After simple algebra, the above equation can be rearranged as follows:

(A8) $${\Delta \rho }_{P}=\frac{6\varepsilon^{2}}{\pi }\sqrt{\frac{N_{agg}I\left(0\right)}{K}}\frac{1}{{X^{2}D}_{M}^{3}}+\frac{R^{2}-R_{H}^{2}}{{X^{2}D}_{M}^{3}\Delta },\;{\Delta \rho }_{P}-{\Delta \rho }_{E}=\frac{R^{2}-R_{H}^{2}}{D_{E}^{3}\Delta },$$

where

(A9) $$∆=\frac{\pi }{120\varepsilon^{4}}\sqrt{\frac{K}{N_{agg}I\left(0\right)}}\left[\left(\varepsilon^{2}+2X^{2}\right)D_{M}^{2}-\left({2+\varepsilon }^{2}\right){\left(D_{M}-2R_{sh}\right)}^{2}\right],\;R_{H}^{2}=\frac{\left(\varepsilon^{2}+2X^{2}\right)D_{M}^{2}}{20\varepsilon^{2}}$$

*R_H_* is the gyration radius for a homogenous elliptical spheroid if the micelle had a constant electron density without any difference between core and shell values. For *R* = *R_H_*, one would have the average electron density within the micelle.

(A10) $$\langle \Delta \rho \rangle =\frac{\sqrt{\frac{N_{agg}I\left(0\right)}{K}}}{V_{M}}=\sqrt{\frac{N_{agg}I\left(0\right)}{K}}\frac{6\varepsilon^{2}}{\pi {X^{2}D}_{M}^{3}}.$$

Equation (A7) relates the two unknown electron density contrasts to $N_{agg}$, *D_M_*, *R_sh_*, *R*, ε and $I\left(0\right)$. For prolate spheroids $D_{M}$ in Equation (A7) should coincide with the maximum distance $D_{max}$ measured in the PDF, i.e., $D_{M}=D_{max}$. For oblate (ε<1) spheroids $D_{M}=\varepsilon D_{max}$. $D_{max}$ and $R_{sh}$ can be determined graphically from the PDF derivative, as discussed in the main section.

*R* can be calculated directly by the PDF curve [17]:

(A11) $$R_{PDF}^{2}=\frac{\int_{0}^{D_{max}}PDF\left(r\right)r^{2}dr}{2\int_{0}^{D_{max}}PDF\left(r\right)dr}.$$

The above equation can be readily generalized for other powers of the distance *r*, leading to Equation (2) of the main text. The quantity *K* in Equations (A8)–(A10), defined by Equation (A4), can be evaluated by the experimental micelle concentration and the properties (molar mass) of the monomer constituting the micelle.

From the analysis of the PDF derivative, discussed in the main section, we have demonstrated that random fluctuations of the shell size affect the half-width of the derivative peak. In turn, this implies that the experimental $R_{PDF}^{2}$ has been affected by these random fluctuations, and, therefore, it is lower than the value corresponding to an ideal shell without any random fluctuation of its size. To estimate the correction factor η that we must apply to the measured gyration ratio, in order to obtain the corresponding value for an ideal micelle without any shell-size random fluctuation, we can average $R_{H}^{2}$ between $D_{M}-$2$R_{sh}$ and $D_{M}$, obtaining $\langle R_{H}^{2}\rangle$, from which:

(A12) $$\eta =\frac{R_{H}^{2}}{\langle R_{H}^{2}\rangle }=\frac{6R_{sh}D_{M}^{2}}{D_{M}^{3}-D_{E}^{3}},$$

Thus, we must multiply the experimental value $R_{PDF}^{2}$, derived by the PDF, by η, in order to derive from Equations (A8) and (A9) reliable electron density contrast’s values for real micelles. Therefore, after some algebra, Equations (A8) and (A9) can be written as follows:

(A13) $${\Delta \rho }_{P}=\langle \Delta \rho \rangle \times \left(1+20\varepsilon^{2}\times \frac{{\eta R}_{PDF}^{2}-R_{H}^{2}}{\left(\varepsilon^{2}+2X^{2}\right)D_{M}^{2}-\left({2+\varepsilon }^{2}\right){\left(D_{M}-2R_{sh}\right)}^{2}}\right),\;{\Delta \rho }_{E}=\langle \Delta \rho \rangle \times \left[1+20\varepsilon^{2}\times \frac{{\eta R}_{PDF}^{2}-R_{H}^{2}}{\left(\varepsilon^{2}+2X^{2}\right)D_{M}^{2}-\left({2+\varepsilon }^{2}\right){\left(D_{M}-2R_{sh}\right)}^{2}}\times \left(1-\frac{{X^{2}D}_{M}^{3}}{D_{E}^{3}}\right)\right],$$

where $R_{H}\;and\langle \Delta \rho \rangle$ are given, respectively, by Equations (A9) and (A10).

The above equations indicate that: ${\Delta \rho }_{P}$ is given by the average electron density value $\langle \Delta \rho \rangle$, within the micelle, adding a positive contribution proportional to the difference of the corrected value of the squared gyration radius ${\eta R}_{PDF}^{2}$ and the value $R_{H}^{2}$ for a homogeneous spheroid of the same micelle’s size; ${\Delta \rho }_{E}$ is given by the same average value $\langle \Delta \rho \rangle$ after substraction of a total positive contribution proportional to the same difference, ${\eta R}_{PDF}^{2}-R_{H}^{2}$.

We still have two unknown quantities to determine, namely *ε* and $N_{agg}$, to obtain all the structural features of the micelle by means of the above equations. Therefore, we need two further independent equations. For this purpose, in the main section, we have already observed that the radius of gyration for a particle with electron density ρ(*r*) is a particular case of the following equation when the integer *n* = 2:

(A14) $$R^{n}=\left(\int r^{n}\left(\rho \left(r\right)-\rho_{s}\right)d^{3}r\right)/\left(\int \left(\rho \left(r\right)-\rho_{s}\right)d^{3}r\right).$$

For positive integers and for a two-component spheroid-shaped micelle, the above integrals have an analytical expression. For *n* = 2, we have the analytical expression for the gyration radius (Equation (A5)). For *n* = 4, we have:

(A15) $$R^{4}=\frac{1}{560\varepsilon^{4}}\times \frac{{\Delta \rho }_{P}X^{2}\left({8X^{4}+4X^{2}\varepsilon }^{2}+{3\varepsilon }^{4}\right)D_{M}^{7}+\left({\Delta \rho }_{E}-{\Delta \rho }_{P}\right)\left({8+4\varepsilon }^{2}{+3\varepsilon }^{4}\right){\left(D_{M}-2R_{sh}\right)}^{7}}{{\Delta \rho }_{P}{X^{2}D}_{M}^{3}+\left({\Delta \rho }_{E}-{\Delta \rho }_{P}\right){\left(D_{M}-2R_{sh}\right)}^{3}}.$$

For *n* = 6, we have:

(A16) $$R^{6}=\frac{1}{6720\varepsilon^{6}}\times \frac{{\Delta \rho }_{P}X^{2}\left({16X^{4}+8X^{4}\varepsilon }^{2}+6X^{2}\varepsilon^{4}+{5\varepsilon }^{6}\right)D_{M}^{9}+\left({\Delta \rho }_{E}-{\Delta \rho }_{P}\right)\left({16+8\varepsilon }^{2}{+6\varepsilon }^{4}{+5\varepsilon }^{6}\right){\left(D_{M}-2R_{sh}\right)}^{9}}{{\Delta \rho }_{P}{X^{2}D}_{M}^{3}+\left({\Delta \rho }_{E}-{\Delta \rho }_{P}\right){\left(D_{M}-2R_{sh}\right)}^{3}}.$$

Both $R^{4}$ and $R^{6}$ can be evaluated by the experimental PDF, as performed by means of Equation (A11) for the gyration radius. We have:

(A17) $$R_{PDF}^{4}=\frac{\int_{0}^{D_{max}}PDF\left(r\right)r^{4}dr}{8\int_{0}^{D_{max}}PDF\left(r\right)dr},$$

(A18) $$R_{PDF}^{6}=\frac{\int_{0}^{D_{max}}PDF\left(r\right)r^{6}dr}{32\int_{0}^{D_{max}}PDF\left(r\right)dr}.$$

By comparing the experimental values given by Equations (A17) and (A18) with the analytical Equations (A15) and (A16), we have two more equations for determining experimentally $N_{agg}$ and ε. For this purpose, we first need to correct the experimental values (Equations (A17) and (A18)) for the shell size’s fluctuations, as performed for the gyration radius. We can put for $R_{PDF}^{n}:$

(A19) $$\eta_{n}=\eta^{n/2},$$

where, for *n* = 2, we have $\eta_{2}=\eta$ given by Equation (A12).

Thus, the next two analytical equations are:

(A20) $$\eta_{4}R_{PDF}^{4}\equiv R^{4}=\frac{{\Delta \rho }_{P}X^{2}\left({8X^{4}+4X^{2}\varepsilon }^{2}+{3\varepsilon }^{4}\right)D_{M}^{7}+\left({\Delta \rho }_{E}-{\Delta \rho }_{P}\right)\left({8+4\varepsilon }^{2}{+3\varepsilon }^{4}\right){\left(D_{M}-2R_{sh}\right)}^{7}}{560\varepsilon^{4}\left[{\Delta \rho }_{P}{X^{2}D}_{M}^{3}+\left({\Delta \rho }_{E}-{\Delta \rho }_{P}\right){\left(D_{M}-2R_{sh}\right)}^{3}\right]},$$

(A21) $$\eta_{6}R_{PDF}^{6}\equiv R^{6}=\frac{{\Delta \rho }_{P}X^{2}\left({16X^{4}+8X^{4}\varepsilon }^{2}+6X^{2}\varepsilon^{4}+{5\varepsilon }^{6}\right)D_{M}^{9}+\left({\Delta \rho }_{E}-{\Delta \rho }_{P}\right)\left({16+8\varepsilon }^{2}{+6\varepsilon }^{4}{+5\varepsilon }^{6}\right){\left(D_{M}-2R_{sh}\right)}^{9}}{6720\varepsilon^{6}\left[{\Delta \rho }_{P}{X^{2}D}_{M}^{3}+\left({\Delta \rho }_{E}-{\Delta \rho }_{P}\right){\left(D_{M}-2R_{sh}\right)}^{3}\right]}.$$

By inserting Equation (A13) into Equation (A20), after some algebra, we have:

(A22) $$N_{agg}=N_{agg,ini}\times \frac{{\left[{560\eta }_{4}R_{PDF}^{4}\varepsilon^{4}\bar{∆}\right]}^{2}}{{\left[{\left(D_{M}-2R_{sh}\right)}^{4}\left({8+4\varepsilon }^{2}{+3\varepsilon }^{4}\right)\left(R_{H}^{2}-{\eta_{2}R}_{PDF}^{2}\right)-D_{M}^{4}\left({8X^{4}+4X^{2}\varepsilon }^{2}+{3\varepsilon }^{4}\right)\left(R_{H}^{2}-{\eta_{2}R}_{PDF}^{2}-\bar{∆}\right)\right]}^{2}},$$

where $N_{agg,ini}$ is a first estimate of the number of monomers constituting the micelle, and

(A23) $$\bar{∆}=\frac{\left(\varepsilon^{2}+2X^{2}\right)D_{M}^{2}-\left({2+\varepsilon }^{2}\right){\left(D_{M}-2R_{sh}\right)}^{2}}{20\varepsilon^{2}}.$$

$N_{agg,ini}$ can be estimated by the maximum hydrophobic chain length [18]:

(A24) $$N_{agg,ini}=\frac{4\pi l^{3}(n_{cs})}{3v(n_{cs})},$$

where $n_{cs}$ is the whole carbon atoms, along the chain length, of the hydrophobic part of the monomer constituting the micelle, and where the alkyl chain length l and the corresponding volume v are given by the Tanford formulae [19]:

(A25) $$l(n_{c})=0.15+0.1265\times n_{c}\;[\mathrm{nm}],\;v(n_{c})=0.0277+0.0269\times n_{c}\;[{\mathrm{nm}}^{3}].$$

Here $n_{c}$ denotes the number of carbon atoms in the chain. From the above relations, it follows that, for common surfactants, the ratio of the volume and the extended length of the alkyl chain tail is a constant, independent of the tail length, equal to 0.021 nm^2^ for a single tail and 0.042 nm^2^ for a double tail. This quantity is fundamental in determining the packing of the monomer part constituting the micelles in their core. For more complex surfactant molecules with a head characterized by a lateral size larger than the tail (a single hydrocarbon chain), the above ratio can assume values ranging from 0.021 to 0.042 nm^2^. A lateral surfactant head size larger than the corresponding value of its tail will influence both its packing in the core and the core-shell interface order, causing the ellipticity of the aggregate when the equilibrium aggregation number would require a spherical shape with a radius larger than the maximum extended hydrophobic chain length.

If the mass density of the monomer constituting the micelle is known, another way to estimate $N_{agg,ini}$ is through the following relation [20]:

(A26) $$N_{agg,ini}=\frac{I\left(0\right)}{I_{mon}}$$

where

(A27) $$I_{mon}=\frac{c_{mon}{r_{e}^{2}MW}_{mon}}{d_{m}^{2}N_{A}}\;\times \;{\left(\rho_{m}-\rho_{s}\right)}^{2}.$$

Here, $\rho_{m}$ and $d_{m}$ are the monomer electron and mass density, respectively. The results obtained by Equations (A24) and (A26) can also be averaged to obtain a more reliable starting estimate.

In micelles, the packing factor of the monomers into the micelles is usually less than 1, i.e., $\langle \Delta \rho \rangle <\rho_{m}-\rho_{s}$. Therefore, Equation (A22) leads to a value $N_{agg}<N_{agg,ini}$.

Also ε needs to be determined, but Equation (A21) is difficult to invert to obtain a simple analytical expression for ε. Moreover, more than one mathematical solution could be obtained. How do you select the most reliable one?

Putting *n* = 1 in Equation (A14), it would give another integral that has an analytical expression:

(A28) $$R^{1}=\frac{3}{16\varepsilon }\times \frac{{\Delta \rho }_{P}X^{2}\left(\varepsilon +X^{2}ArcCos\left[\varepsilon /X\right]/\sqrt{X^{2}-\varepsilon^{2}}\right)D_{M}^{4}+\left({\Delta \rho }_{E}-{\Delta \rho }_{P}\right)\left(\varepsilon +ArcCos\left[\varepsilon \right]/\sqrt{1-\varepsilon^{2}}\right){\left(D_{M}-2R_{sh}\right)}^{4}}{{\Delta \rho }_{P}{X^{2}D}_{M}^{3}+\left({\Delta \rho }_{E}-{\Delta \rho }_{P}\right){\left(D_{M}-2R_{sh}\right)}^{3}}.$$

It can be readily verified that Equation (A28) for oblate spheroids is a real quantity. For prolate spheroids, it is a complex quantity with a null imaginary component.

In analogy to the other integrals of the PDF, we can obtain from Equation (A14) the average micelle radius:

(A29) $$R_{PDF}^{1}=\frac{\int_{0}^{D_{max}}PDF\left(r\right)rdr}{\int_{0}^{D_{max}}PDF\left(r\right)dr}.$$

Therefore, another analytical equation is obtained:

(A30) $$\eta_{1}R_{PDF}^{1}=\frac{3}{16\varepsilon }\times \frac{{\Delta \rho }_{P}X^{2}\left(\varepsilon +X^{2}ArcCos\left[\varepsilon /X\right]/\sqrt{X^{2}-\varepsilon^{2}}\right)D_{M}^{4}+\left({\Delta \rho }_{E}-{\Delta \rho }_{P}\right)\left(\varepsilon +ArcCos\left[\varepsilon \right]/\sqrt{1-\varepsilon^{2}}\right){\left(D_{M}-2R_{sh}\right)}^{4}}{{\Delta \rho }_{P}{X^{2}D}_{M}^{3}+\left({\Delta \rho }_{E}-{\Delta \rho }_{P}\right){\left(D_{M}-2R_{sh}\right)}^{3}}.$$

$\eta_{1}$ in the above equation is obtained by inserting *n* = 1 in Equation (A19).

By means of these two further equations—Equation (A28) and Equation (A30)—it is possible to define a Figure of Merit (FOM) useful to select the more reliable value of ε, between the possible physical solutions:

(A31) $$FOM[\varepsilon ]={Abs\left({FOM}_{1}\times {FOM}_{6}-1\right)}^{1/2},$$

where

(A32) $${FOM}_{1}=\frac{{3\Delta \rho }_{P}X^{2}\left(\varepsilon +X^{2}ArcCos\left[\varepsilon /X\right]/\sqrt{X^{2}-\varepsilon^{2}}\right)D_{M}^{4}+3\left({\Delta \rho }_{E}-{\Delta \rho }_{P}\right)\left(\varepsilon +ArcCos\left[\varepsilon \right]/\sqrt{1-\varepsilon^{2}}\right){\left(D_{M}-2R_{sh}\right)}^{4}}{\eta_{1}R_{PDF}^{1}16\varepsilon \left[{\Delta \rho }_{P}{X^{2}D}_{M}^{3}+\left({\Delta \rho }_{E}-{\Delta \rho }_{P}\right){\left(D_{M}-2R_{sh}\right)}^{3}\right]},$$

(A33) $${FOM}_{6}=\frac{{\Delta \rho }_{P}X^{2}\left({16X^{4}+8X^{4}\varepsilon }^{2}+6X^{2}\varepsilon^{4}+{5\varepsilon }^{6}\right)D_{M}^{9}+\left({\Delta \rho }_{E}-{\Delta \rho }_{P}\right)\left({16+8\varepsilon }^{2}{+6\varepsilon }^{4}{+5\varepsilon }^{6}\right){\left(D_{M}-2R_{sh}\right)}^{9}}{\eta_{6}R_{PDF}^{6}6720\varepsilon^{6}\left[{\Delta \rho }_{P}{X^{2}D}_{M}^{3}+\left({\Delta \rho }_{E}-{\Delta \rho }_{P}\right){\left(D_{M}-2R_{sh}\right)}^{3}\right]}.$$

${FOM}_{1}$ depends linearly on distances. ${FOM}_{6}$ depends on a 6-th power of distances. Therefore, the FOM defined in Equation (A31) is simultaneously dependent on both the lowest and largest distances in the PDF. Thus, to find the best experimental ellipticity’s value of the micelles under study, we could change numerically the value of ε, usually ranging from 0.5 to 2.0, until the value in Equation (A31) of FOM[ε] reaches a minimum, i.e., until Equations (A28) and (A30) are satisfied. In this way, the solution for the ellipticity is constrained by more than one equation, derived from the integrals of the experimental PDF, thus giving a more reliable solution about all possible oblate-elliptical/prolate-elliptical/spherical-shape alternatives.
